# Associations between high callous–unemotional traits and quality of life across youths with non-conduct disorder diagnoses

**DOI:** 10.1007/s00787-015-0766-5

**Published:** 2015-09-11

**Authors:** Pierre C. M. Herpers, Helen Klip, Nanda N. J. Rommelse, Corina U. Greven, Jan K. Buitelaar

**Affiliations:** University Center, Karakter Child and Adolescent Psychiatry, Reinier Postlaan 12, 6525 GC Nijmegen, The Netherlands; Department of Psychiatry, Donders Institute for Brain, Cognition and Behaviour, Radboud University Medical Center, Nijmegen, The Netherlands; Department of Cognitive Neuroscience, Donders Institute for Brain, Cognition and Behaviour, Radboud University Medical Center, Nijmegen, The Netherlands; Institute of Psychiatry, Psychology & Neuroscience, Medical Research Council Social, Genetic & Developmental Psychiatry Centre, King’s College London, London, UK

**Keywords:** Callous–unemotional traits, ADHD, ASD, Mood disorder, Conduct disorder, Quality of life

## Abstract

Research regarding callous–unemotional (CU) traits in non-conduct disorder (CD) diagnoses is sparse. We investigated the presence of high CU traits and their associations with quality of life (QoL) in a clinically referred sample of youths with non-CD diagnoses. Parents of 1018 children referred to a child and adolescent psychiatric clinic and rated their child’s CU traits and QoL. Experienced clinicians derived DSM-IV-TR diagnoses based on systematic clinical evaluations of these children. High CU traits compared to low CU traits were present in 38.5 % of the sample, and more often in boys than girls (69.4 vs. 30.6 %, *p* = .004), and were associated with more police contacts (12.2 vs. 3.5 %, *p* < .001). Logistic regression analyses revealed that those with diagnoses of autism spectrum disorder (odds ratio; OR = 1.61; 95 % CI 1.24–2.09; *p* < .001) and disruptive behavior disorder not otherwise specified/oppositional defiant disorder (OR = 4.98; 95 % CI 2.93–8.64; *p* < .001), but not attention-deficit/hyperactivity disorder (OR = 1.01; 95 % CI .79–1.31; *p* = .94), were more likely to have high than low CU traits. Those with anxiety/mood disorders were more likely to have low than high CU traits (OR = .59; 95 % CI .42–82; *p* = .002). In all diagnostic groups, high CU compared to low CU traits were associated with significantly lower QoL, while controlling for gender, age, and comorbidity. As such, high CU traits significantly modify QoL in non-CD disorders.

**Electronic supplementary material:**

The online version of this article (doi:10.1007/s00787-015-0766-5) contains supplementary material, which is available to authorized users.

## Introduction

Callous–unemotional (CU) traits are thought to represent the core component of psychopathy, and include symptoms such as lack of feeling guilty, lack of empathy, being very egocentric, showing callous use of others for one’s own gain, and lacking normal emotionality, especially in showing a lack of anxiety [13]. CU traits were incorporated in the DSM-5 [2] as a specifier for conduct disorder (CD). The specifier consists of four criteria of which at least two should be met to index a more severe form of CD. CU traits in children with conduct problems (CP) have been reported to imply increased levels of aggressive behaviors, worse prognosis, and treatment refractoriness [14]. Increasingly, research shows neurobiological underpinnings of psychopathy, in which reduced amygdala responsiveness to distress cues results in reduced empathic response to distress of other individuals (as captured by the callous–unemotional component of psychopathy). In addition, dysfunction in the ventromedial prefrontal cortex and striatum results in deficient decision making and reinforcement learning (as captured by the impulsive–antisocial component of psychopathy; for a comprehensive review, see [5]).

The majority of research on CU traits has been conducted in youths with CP. However, little is known about the presence of CU traits in disorders other than CD and about their implications for severity of these disorders and functional adaptation [20, 32]. In previous research, CU traits were not associated with quality of life (QoL) in a clinical sample of youths with CD [22]. Impairment in youths with CP showed either no [19, 22, 29, 30] or a positive [15, 25] relationship with CU traits. In community samples high CU traits were associated with more global impairment [10, 34, 43], not only in the CD subsample, but in the no CD/high CU subsample as well [34]. There are indications that impairment in the presence of attention-deficit/hyperactivity disorder (ADHD) symptoms may be moderated by CU traits [6, 44]. However, no studies reported yet on QoL in specific non-CD diagnoses.

Our cross-sectional study extends previous research, by examining associations between CU traits and non-CD diagnoses in a large clinical sample and by investigating relations between CU traits and QoL across non-CD disorders. We addressed the following questions: (1) what are the odds for scoring high on CU traits when being diagnosed having a psychiatric disorder other than CD?, (2) what is the relation between high CU traits and QoL in non-CD diagnoses?

## Method

### Sample

Data were used of a sample of 1833 juveniles (aged 6 and older) who were consecutively referred to Karakter, a child and adolescent psychiatric clinic in the Netherlands. We focused on data collected between July 2012 and May 2013. Services ranged from outpatient to high intensive mental healthcare, for patients with an estimated normal intelligence (IQ >70). Estimated intelligence is based on either clinical functioning (e.g., in case of good functioning in school) or by administering an intelligence test (i.e., predominantly the Wechsler Intelligence Scale for Children [45]). Clinical DSM-IV-TR [3] diagnoses were established by a multidisciplinary team based on information gathered by a child psychiatrist (developmental history, child observation, and psychiatric assessment), by a child psychologist, and review of clinical and prior records, including information available from school or other professional institutions involved with the child. Thus, a consensus diagnosis is assigned, which is seen as most reliable, compared to structured interviews when broad diagnostic categories are investigated [27]. In The Netherlands, severe CP are usually not treated within a psychiatric setting, but in juvenile welfare centers or juvenile penitentiary institutions. Hence, our clinic serves a specific population in which disruptive behavior disorders are only seen as a comorbid disorder and not as primary diagnosis.

Before the first appointment at the clinic, parents completed a digital intake questionnaire which assessed a range of variables, including age, gender, country of birth, number of police contacts of the child, education level of parents, and also included validated questionnaires such as the Kidscreen-27 [37] for measuring QoL. For this study, the Inventory of Callous–Unemotional traits (ICU) [12] was added to the intake questionnaire. Global functioning was rated by experienced child and adolescent psychiatrists using the Global Assessment of Functioning Scale according the DSM-IV-TR [3] criteria. Scores above 60 reflect no or minor functional impairment. This study was approved by the Institutional Review Board.

Participants whose parents gave informed consent (*n* = 1190) were included in this study. However, at the time of statistical analysis, data regarding DSM-IV-TR diagnosis were missing for *n* = 154, in which additional diagnostic information was being gathered and consensus was not reached yet. Furthermore, 7 juveniles were excluded because of invalid entry of Kidscreen scores (described below), 6 juveniles because of age above 18 years, and 5 juveniles because they were diagnosed with CD. This resulted in a sample of 1018 youths.

### Measures

Parents rated their child on *callous*–*unemotional traits* using the Dutch translation of the 24-item ICU, which assesses callous–unemotional personality traits [39], rated on a 4-point Likert scale ranging from 0 = *does not apply at all* to 3 = *applies very well*. Internal consistency of the Dutch ICU was shown to be good [8, 39]. In our study Cronbach’s Alpha was also good (.89). Concurrent validity between the ICU and psychopathy scales is acceptable (*r*^2^ = .45–.68) between ICU and antisocial process screening device (APSD), and childhood psychopathy scale (CPS) [24, 39]. Mean total score for the parent report ICU in a community sample (aged 11–16 years, *n* = 174) was previously found to be 22.28 (SD = 9.30; [26]).

*Quality of life* was measured by parent ratings on the Kidscreen-27 [36]. The Kidscreen-27 assesses general well-being and subjective health in youths, and contains 27 questions related to five dimensions (Physical Well-being, Psychological Well-being, Autonomy and Parent Relations, Social Support and Peers, School Environment), as well as giving rise to a total QoL score. Each item is being rated on a 5-point Likert scale from 1 = *not at all/never* to 5 = *totally/always*. Thus, low Kidscreen scores reflect lower QoL. Previous studies showed a positive relationship between severity of mental health problems in youths and QoL as measured by the Kidscreen-27 [9, 46]. The questionnaire has been tested in multiple countries and is validated in Dutch. Internal consistencies (Cronbach’s Alpha) were reported to be >0.75 [36]. In our study, Cronbach’s Alpha for the total Kidscreen-27 was .90.

### Analyses

IBM SPSS Statistics 21 was used for statistical analyses. The continuous data on the ICU were broken down into a high and a low scoring group. Although no widely accepted cut-off has been accepted yet, we used a cut-off score of 32 on the ICU (i.e., score <32 = low CU; score ≥32 = high CU). This is in line with previous studies [21, 41], in which youths with similar age, with CP, and also with autism spectrum disorder (ASD) was included. In our sample, the mean ICU score was 28.75 (SD = 11.21), which is similar to the mentioned studies. We made use of five diagnostic categories: ADHD, ASD, anxiety/mood, disruptive behavior disorder not otherwise specified (DBD-NOS)/ODD, and other diagnoses (see also Table S1, available online). Information on comorbidity is given in Table S2 (available online). Bivariate correlations are shown in Table S3 (available online). Diagnostic groups with high versus low CU traits were compared on sample descriptives using *χ*^2^ tests. To study the associations between diagnosis and presence of high CU traits, logistic regression analyses were performed entering diagnosis as dependent dichotomous variable and high/low ICU score as independent categorical variable. We repeated these analyses including age and gender as covariates. To examine whether high CU traits were linked to QoL, *t* tests were performed to analyze the relationship between the dichotomous ICU scores and continuous Kidscreen data for the total sample and for each diagnostic group. Second we performed hierarchical multiple linear regression analyses predicting Kidscreen-27 scores (continuous variable) from age, gender (entered in step 1), and the dichotomous ICU variable (entered in step 2) for each diagnostic group. We have rerun analyses with exclusion of those participants whose IQ level was estimated being below 85.

To test the robustness of our findings versus the chosen cut-off of 32, analyses were rerun with slightly higher or lower cut-offs (i.e., ICU ≥28 and ICU ≥35) in addition to a hierarchical multiple linear regression with the continuous ICU score. We ran primary analyses on groups including comorbidities to make use of the full sample; however, we also reran analyses on participants free of any comorbidity to examine potential confounding by comorbidity. We also ran sensitivity analyses to test the robustness of results. First, we examined whether results were similar for continuous ICU scores. Second, we examined the role of IQ by excluding those with an estimated IQ below 85. Third, we examined the role of stressful life events, measured as binary variable (yes/no), as reported by parents.

## Results

### Descriptive results of the study sample by high and low CU traits

The characteristics of the total sample, and separately for youths with high versus low CU traits are described in Table 1. The high scoring group (i.e., ICU total score ≥32) consisted of 392 participants, and the low scoring group of 626 participants. Mean ICU score for the high CU group was 40.26 (SD = 6.83). Mean ICU score for the low CU group was 21.54 (SD = 6.32). Children scoring high on CU traits were more often male than female (69.4 vs. 30.6 %, *p* = .004). Age, living in a larger city (≥100,000 inhabitants), and education level of the parent showed no significant correlations with CU traits. In addition, we found that those with high CU traits had lower Global Assessment of Functioning (GAF) scores (*p* = .001) and more police contacts (12.2 vs. 3.5 %, *p* < .001) than those scoring low on CU traits.

**Table 1:** Characteristics of the study population, for the total group and for the ICU groups (*N* = 1018)

		Total group (*n* = 1018)		ICU				*p* value^a^
				ICU score <32 (*n* = 626)		ICU score ≥32 (*n* = 392)		
		*n*	%	*n*	%	*n*	%	
Age (years)								
6 ≤ 11		572	56.2	366	58.5	206	52.6	.11
12 ≤ 15		330	32.4	188	30.0	142	36.2	
16 ≤ 18		116	11.4	72	11.5	44	11.2	
Gender								
Male		651	63.9	379	60.5	272	69.4	.004
Female		367	36.1	247	39.5	120	30.6	
Education level of child								
Primary education		404	42.0	275	46.4	129	35.0	.001
Special needs education		177	18.4	100	16.9	77	20.9	
Special needs secondary education		108	11.2	57	9.6	51	13.8	
Preparatory middle-level vocational education		142	14.8	74	12.5	68	18.4	
Higher vocational education/preparatory university education		131	13.6	87	14.7	44	11.9	
City ≥100.000								
Yes		308	30.4	190	30.4	118	30.3	.97
Education level of parent								
Lower		134	13.8	79	13.2	55	14.9	.75
Middle		415	42.9	259	43.2	156	42.3	
Higher		419	43.3	261	43.6	158	42.8	
GAF score								
1 ≤ 40		113	11.1	58	9.3	55	14.0	<.001
41 ≤ 60		832	81.8	508	81.3	324	82.7	
61 ≤ 100		72	7.1	59	9.4	13	3.3	
Police contacts								
Yes		70	7.2	22	31.4	48	68.6	<.001
Stressful life events								
Yes		563	59.3	334	59.3	229	40.7	<.05

*GAF* global assessment of functioning

^a^ *χ* ^2^ test comparing ICU score <32 vs. ICU score ≥32 on study population characteristics

### Odd ratios for scoring high on CU traits in non-CD youths with psychopathology

Of the total sample, 38.5 % scored high on CU traits. In Table 2 the mean ICU scores per diagnosis are shown. Diagnoses of ASD (odd ratio; OR = 1.61; 95 % CI 1.24–2.09; *p* < .001) and DBD-NOS/ODD (OR = 4.98; 95 % CI 2.93–8.64; *p* < .001), but not ADHD (OR = 1.01; 95 % CI .79–1.31; *p* = .94), were more often associated with the presence of high than low CU traits. Anxiety/mood disorders were more often associated with low than with high CU traits (OR = .59; 95 % CI .42–82; *p* = .002). For the ADHD and the other diagnoses groups, the OR was not significantly elevated. Entering age and gender into the model, for all diagnostic groups, the ORs were similar in magnitude and remained significant for ASD, DBD-NOS/ODD, and anxiety/mood disorders.

**Table 2:** Associations between diagnosis and risk for ICU ≥32 expressed as odds ratio, and prevalence of high CU scores

Diagnosis^a^	ICU score		OR	95 % CI	OR_adj_	95 % CI_adj_	ICU <32		ICU ≥32	
	*M*	SD					*n*	%	*n*	%
ADHD	28.7	11.0	1.02	.79–1.31	1.01	.79–1.31	274	61.3	173	38.7
ASD	30.6	10.9	1.68**	1.30–2.17	1.61**	1.24–2.09	238	54.5	199	45.5
Anxiety/mood	26.2	11.5	.61*	.45–0.83	.59*	.43–.82	167	70.2	71	29.8
DBD-NOS/ODD	37.6	11.7	5.05**	2.98–8.56	4.98**	2.93–8.47	20	26.3	56	73.7
Other diagnoses	29.2	11.6	1.08	.84–1.40	1.11	.86–1.44	256	60.4	168	39.6
Total group	28.8	11.2					626	61.5	392	38.5

*adj* adjusted for age and gender, *OR* odds ratio, *ICU* inventory of callous–unemotional traits, *95* *% CI* 95 % confidence interval, *ADHD* attention-deficit/hyperactivity disorder, ASD autism spectrum disorder, *Anxiety/mood* either anxiety or mood disorder, *DBD-NOS/ODD* either disruptive behavior disorder not otherwise specified or oppositional defiant disorder, *Other Diagnoses* are listed in table S1

* *p* = .002; ** *p* < .001

^a^With any or without comorbidity

### High CU traits and their relation to QoL in non-CD youths with psychopathology

**Fig. 1:**
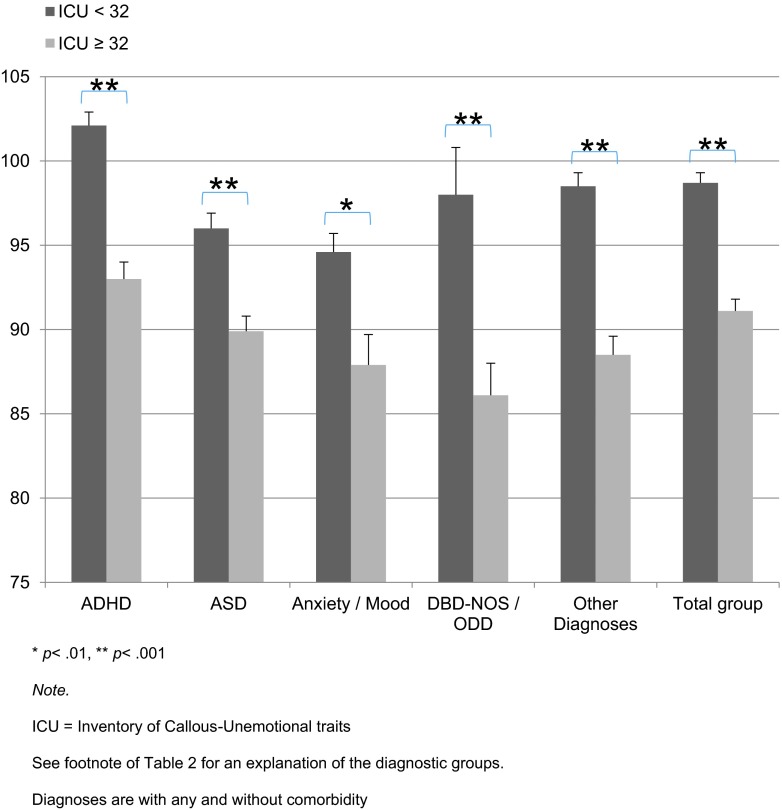
Mean Kidscreen scores and standard error of the mean (SEM) by diagnosis, *p < .01, **p < .001, *ICU* inventory of callous–unemotional traits, *ADHD* attention-deficit/hyperactivity disorder, ASD autism spectrum disorder, *Anxiety/mood* either anxiety or mood disorder, *DBD-NOS/ODD* either disruptive behavior disorder not otherwise specified or oppositional defiant disorder, *Other Diagnoses* are listed in table S1. Diagnoses are with any and without comorbidity

The Kidscreen analyses for the total sample showed that Kidscreen scores in the high CU group were significantly lower than those in the low CU group (91.1 vs. 98.7, *p* < .001; see Fig. 1). Similar findings emerged for all specific diagnostic groups: the high CU group showed lower Kidscreen scores for ADHD (92.9 vs. 102.1, *p* < .001), ASD (89.9 vs. 95.9, *p* < .001), anxiety/mood (87.9 vs. 94.6, *p* = .002), DBD-NOS/ODD (86.1 vs. 98.3, *p* < 0.001), and other diagnoses (91.1 vs. 98.5, *p* < .001).

High ICU scores predicted Kidscreen scores (*β*_Total group_ = −.266, *p* < .001; *β* = standardized regression coefficient), explaining 4–13 % of the variance in Kidscreen scores beyond effects of age and gender (see Table 3). Collinearity statistics showed that the results for the variance inflation factor in the linear regression analyses (taken the analyses with and those without comorbidity together) ranged between 1.001 and 1.126, while tolerance ranged between .888 and .999. Thus, there seems to be no collinearity. Post hoc analyses entering age, gender, education level of parents, police contacts, and DBD-NOS/ODD in step 1 of the regression and ICU scores in step 2 found that education level of parents and the police contacts did not contribute significantly to the model, while DBD-NOS/ODD contributed significantly in the total group (*β* = .061, *p* < .05), in the ASD (*β* = .133, *p* < .01), and in the anxiety/mood disorders group (*β* = .131, *p* < .05). However, results for CU traits stayed similar.

**Table 3:** Hierarchical multiple linear regression analyses predicting Kidscreen scores from age, gender, and ICU by diagnosis

	Total group		ADHD^a^		ASD^a^		Anxiety/mood^a^		DBD-NOS/ODD^a^		Other diagnoses^a^	
	Δ*R* ^2^	*β*	Δ*R* ^2^	*β*	Δ*R* ^2^	*β*	Δ*R* ^2^	*β*	Δ*R* ^2^	*β*	Δ*R* ^2^	*β*
Step 1	.076***		.055***		.043***		.072***		.104*		.123***	
Age		−.252***		−.230***		−.190***		−.195**		−.323**		−.278***
Gender		−.084**		−.033		−.078		−.153*		.012		−.170***
Step 2	.070***		.098***		.053***		.041**		.125**		.092***	
ICU ≥ 32		−.266***		−.315***		−.230***		−.205**		−.359**		−.307***
Total *R* ^*2*^	.147***		.153***		.096***		.114**		.229**		.215***	
*n*	1014		444		434		237		73		421	

*β* standardized regression coefficient, *ICU* inventory of callous–unemotional traits, *ADHD* attention-deficit/hyperactivity disorder,* ASD* autism spectrum disorder, *Anxiety/mood* either anxiety or mood disorder, *DBD-NOS/ODD* either disruptive behavior disorder not otherwise specified or oppositional defiant disorder, *Other Diagnoses* are listed in table S1

* *p* < .05, ** *p* < .01 *** *p* < .001

^a^With any or without comorbidity

### Sensitivity analyses

Bivariate correlations with continuously distributed ICU scores (Table S3) yielded a significant inverse relationship between the continuous ICU and Kidscreen-27 scores (*r* = −.32, *p* < .001), and continuous ICU and GAF scores (*r* = −.16, *p* < .001). Regression analyses yielded similar results when we entered ICU continuous scores into the model (Table S5, available online) or when using slightly higher or lower cut-off scores (i.e., ICU ≥28 and ICU ≥35). In the ‘no co-morbidity’ analyses, most results stayed in the same direction. Nevertheless, the group with ADHD only showed a decrease of the likelihood of scoring high on CU traits, while findings for the other groups were in the same direction as in the primary analyses including comorbidities (see Table S4, available online). Results of the regression analyses no longer reached significance in the anxiety/mood and the DBD-NOS/ODD group (see Tables S6 and S7, available online). Rerunning our analyses excluding those participants with an estimated IQ below 85, overall, results stayed similar and significant for the ORs. For the regression analyses, results stayed in the same direction. Finally, *χ*^2^ analyses showed stressful life events to be significantly more present in youths with high CU traits. However, bivariate correlations did show only small, non-significant relationships. Adding stressful life events to the first step of our regression analyses did not alter results regarding the ICU, except in the other diagnoses group, in which significance of the predictive value of the ICU was lost.

## Discussion

This study investigated whether CU traits should be seen as a cross disorder phenomenon that also occurs outside CD, and whether high CU traits outside CD affect functioning in important domains as reflected in a measure of QoL. In our large clinical sample, the odds for high CU traits were found to be significantly increased in ASD and DBD-NOS/ODD, while the odds for high CU traits were found to be significantly decreased in anxiety/mood disorders. For ADHD and other diagnoses, the odds for high CU traits were not significantly increased. A new and important finding is that, in all diagnostic groups, high CU traits were associated with lower QoL, and explained a significant proportion of variance (4–13 %) in QoL beyond effects of age and gender. In contrast, education level of child or parents and the police contacts were unrelated to QoL.

These findings support and extend earlier studies reporting on CU traits being present outside CD [40] and on the negative impact of CU traits across disorders [7]. In contrast to previous research in youths with CP (which refers to a broader concept than our DBD-NOS/ODD diagnosis), we found QoL to be significantly decreased in the high CU group compared to the low CU group. Our DBD-NOS/ODD only sample was small, however, findings remained in the same direction when controlling for comorbidity. Thus, our results underscore the importance of considering CU traits as a specifier for ODD as well [20]. Nevertheless, as only one scale and only one source of information has been used to detect CU traits, we need to keep in mind that parents might rate ‘Has your child been able to pay attention’ (Kidscreen-27 item) similar as being ‘concerned about schoolwork’ (ICU item).

In the ADHD group, our findings contrast with previous results showing that CU traits moderate functional impairment in those with low and moderate levels of ADHD symptoms [6]. In our sample, CU traits were related to QoL in those with a diagnosis of ADHD, all of whom had at least moderate or high levels of ADHD symptoms. This is an important finding because CU traits may moderate treatment response in ADHD as well. Effects of behavioral therapy were found to be less in the presence of high CU traits compared to those with low CU traits [16–18, 42], which might be related to the component of punishment in the treatment program [31]. Methylphenidate was found to show a positive effect on CU traits [4] and on CP [42]. However, it is unclear whether the effect of CU traits in these ADHD samples was confounded by comorbidity with ODD or CD. Therefore, we controlled for comorbid DBD-NOS/ODD and were able to show CU traits having incremental value in predicting QoL in youths with ADHD, over and beyond CP. It is important to note that this effect existed independently of the finding that on average there was no increased OR for CU traits in ADHD. Thus, CU traits may be an independent predictor of treatment response regardless of diagnosis.

Previous studies showed that high scores on CU traits in youths with ASD may, at least in part, reflect theory of mind (ToM) deficits due to impaired empathic response to distress cues [38], cognitive empathy deficits [21, 41], and significantly decreased medial prefrontal cortex responses during ToM tasks in youths with ASD compared to youths with CP and high CU traits, and typically developing youths [33]. Thus, high CU traits in ASD may be due to different underlying cognitive and brain mechanisms than high CU traits in CD. As we found a significantly increased OR for CU traits in ASD in this clinical sample, and high CU traits negatively impacted upon QoL, it is important to further unravel the relationship between CU traits and ASD.

This study confirmed previous studies that found a negative relationship between anxiety and CU traits (for a review, see e.g., [14]). The relationship between high CU traits and mood disorders has been investigated scarcely (see e.g., [20]). We found high CU traits within anxiety/mood disorders to be related to significantly lower QoL. In this diagnostic group, this effect existed independently of the finding that on average there was a decreased OR for CU traits. However, when controlling for comorbidity, this effect disappeared, which might be due to the relative small number of participants in the high CU group (9 versus 39 in the low CU group). Also important to note is the fact that it is unknown whether CU traits in anxiety/mood disorders represent the same underlying construct as in youths with CP. Parents might, for example, be rating anhedonia rather than the lack of emotion, or social avoidance rather than hiding one’s emotions. Further investigation of our finding is therefore needed in either anxiety and mood disorder only groups, as well as further research into the prognosis and treatment results of high CU traits in the presence of anxiety/mood disorders.

The main strength of this study was that it focused on the relation between CU traits and QoL in clinically established non-CD diagnoses in a large psychiatric outpatient sample. However, an important limitation is the lack of control groups, such as either (a large group of) youths with disruptive behavior disorders and normal controls. A potential limitation is the fact that there is no established cut-off score for the ICU. Although it still is difficult to define which is the best way to establish cut-off scores regarding the ICU [23], our results give important information about the distribution of high CU traits across non-CD disorders. Furthermore, the fact that we relied on parent-reported assessment of CU traits and of QoL may mean that shared method variance may have inflated correlations between these two variables. However, regarding the individuals items, there is virtually no overlap, which also emerged from collinearity analyses. Nevertheless, future studies might adopt a more comprehensive multi-informant multi-measure approach to assess CU traits, to be able to address issues of potential informant and instrument bias. Also, future research might benefit a structured interview to establish CU traits, such as the Clinical Assessment of Prosocial Emotions [11] which provides DSM diagnoses of CU traits. However, this is a clinician-rated measure and published only recently, and not available at time of our data collection. However, its value has to be established yet. Similarly, much of the analyses depend on clinical diagnoses that were not made with a structured and well-established clinical interview (e.g., the K-SADS). Nevertheless, our diagnostic procedures were similar to those to reach best estimate clinical diagnoses and as such, thought to be more reliable than the use of structured interviews only [27]. Not having addressed intelligence as a covariate in our analyses is also a limitation. As we did not gather information regarding intelligence in a standardized way, we applied a broad definition of intelligence in terms of identifying individuals with borderline intellectual functioning (IQ below 85). Although our results stayed similar when excluding those with an estimated IQ below 85, previous findings about the relationship between psychopathic traits and intelligence in conduct disorder have been contrasting [1]. Therefore, it could be noteworthy to specifically examine the impact of IQ on CU traits in non-CD disorders. Also, we did not investigate the temporal relation between CU traits and stressful life events. Given the discussion regarding secondary psychopathy in which traumatization is seen as possible moderating CU traits [28], it may be important to investigate whether traumatized children show CU behavior due to their traumatization (‘emotional numbness’), rather than having a truly underlying high CU phenotype. As we did not gather longitudinal data, we have no information that addresses a developmental perspective of CU traits, and as such its possible malleability through time [35].

Several unanswered questions remain for future research. One such question is: what is the mechanism through which high CU traits affect QoL in non-CD disorders? The relationship between CU traits and non-CD disorders is poorly understood. It is unknown how peers perceive youths with high CU traits, and there may be other ways in which social relationships are compromised. Also we do not know whether there is equifinality or multifinality in the causes of high CU traits in non-CD disorders compared to those causes in CD. High CU traits may be related to decreased problem solving skills and thus predispose to less help-seeking behavior or being less help receptive and thus to decreased QoL. Although age was not found to be a significant moderator or covariate, it remains unclear whether CU traits in these diagnostic groups may have different meanings at different ages. As such, many issues for further research remain.

## Conclusion

This study showed that increased CU traits are not limited to CD only. Instead, CU traits occur as a cross disorder phenomenon, and are related to low QoL even in disorders that are not per se associated with an increased risk for high CU traits. Our data suggest clinicians should pay attention to CU traits also in non-CD disorders. Further research is needed into the consequences of high CU traits for response to treatment, prognosis, and course of these non-CD disorders.

## Electronic supplementary material

Below is the link to the electronic supplementary material.

Supplementary material 1 (DOCX 46 kb)
