# Supplementary material for: Associations between high callous–unemotional traits and quality of life across youths with non-conduct disorder diagnoses
Source: Eur Child Adolesc Psychiatry. 2015 Sep 11;25:547–55. doi: 10.1007/s00787-015-0766-5 (PMC4854931; doi:10.1007/s00787-015-0766-5)
Supplement: Supplementary file 1 — Supplementary material 1 (DOCX 46 kb) [file 787_2015_766_MOESM1_ESM.docx]

**Appendix for online-only version: Supplementary material**

Supplementary material may be found in the online version of this article:

**Table S1**

Diagnoses listed as ‘other diagnoses’

**Table S2**

Diagnoses in the study population (N=1,018)

**Table S3**

Bivariate correlations

**Table S4**

Associations between diagnosis and risk for ICU ≥ 32 expressed as odds ratio, and prevalence of high CU scores

**Table S5**

Hierarchical multiple linear regression analyses predicting Kidscreen scores from age. gender and ICU total score (continuous) by diagnosis

**Table S6**

Mean Kidscreen scores and standard error of the mean (SEM) by diagnosis

**Table S7**

Hierarchical multiple linear regression analyses predicting Kidscreen scores from age, gender and ICU by diagnosis

Table S1

*Diagnoses listed as ‘other diagnoses’*

| Academic problem |
| --- |
| Adjustment disorder |
| Cognitive disorder |
| Communication disorder |
| Developmental coordination disorder |
| Disorder of infancy, childhood, or adolescence not otherwise specified |
| Elimination disorder |
| Gender identity disorder |
| Impulse-control disorder |
| Identity problem |
| Learning disorder |
| Mental disorder due to a general medical condition |
| Narcolepsy |
| Phase of live problem |
| Physical abuse of child |
| Pica |
| Primary insomnia |
| Psychotic disorders |
| Unspecified mental disorder (nonpsychotic) |
| Reactive attachment disorder of infancy or early childhood |
| Relational problem |
| Selective mutism |
| Sleeping disorder |
| Stuttering |
| Tic disorder |
| Neglect of child |
| Feeding and eating disorders of infancy or early childhood |

Table S2

*Diagnoses in the study population (N=1,018)*

| Diagnosis | Absent | |  | Present | | | | | | | | | | | | | | | |
| --- | --- | --- | --- | --- | --- | --- | --- | --- | --- | --- | --- | --- | --- | --- | --- | --- | --- | --- | --- |
|  |  | |  | Without comorbidity | |  | With any comorbidity | | | | | | | | | |  | Total^2^ | |
|  |  | |  |  |  |  | ADHD^1^ | | ASD^1^ | | Anxiety / Mood^1^ | | DBD-NOS / ODD^1^ | | Other^1^ | |  |  | |
|  | *n* | % |  | *n* | % |  | *n* | % | *n* | % | *n* | % | *n* | % | *n* | % |  | *N* | % |
| ADHD | 571 | 56 |  | 166 | 16 |  |  |  | 124 | 12 | 61 | 6 | 39 | 4 | 157 | 15 |  | 447 | 44 |
| ASD | 581 | 57 |  | 205 | 20 |  | 124 | 12 |  |  | 48 | 5 | 20 | 2 | 122 | 12 |  | 437 | 43 |
| Anxiety / Mood | 780 | 77 |  | 48 | 5 |  | 61 | 6 | 48 | 5 |  |  | 9 | 1 | 124 | 12 |  | 238 | 23 |
| DBD-NOS / ODD | 942 | 93 |  | 13 | 1 |  | 39 | 4 | 20 | 2 | 9 | 1 |  |  | 35 | 3 |  | 76 | 7 |
| Other Diagnoses | 594 | 58 |  | 76 | 8 |  | 157 | 15 | 122 | 12 | 124 | 12 | 35 | 3 |  |  |  | 424 | 42 |

^1^ Numbers do not add up since patients can have more than one comorbidity

^2^ With any or without comorbidity

*Note.*

ADHD = Attention-Deficit/Hyperactivity Disorder

ASD = Autism Spectrum Disorder

Anxiety / Mood = either Anxiety or Mood disorder

DBD-NOS / ODD = either Disruptive Behavior Disorder Not Otherwise Specified or Oppositional Defiant Disorder

Other Diagnoses = diagnoses listed in table S1

Table S3

*Bivariate correlations*

|  |  | 1 | 2 | 3 | 4 | 5 | 6 | 7 | 8 | 9 | 10 | 11 | 12 | 13 | 14 |
| --- | --- | --- | --- | --- | --- | --- | --- | --- | --- | --- | --- | --- | --- | --- | --- |
| 1 | ICU total^a^ | 1 |  |  |  |  |  |  |  |  |  |  |  |  |  |
| 2 | Kidscreen-27 Total^a^ | -.32*** | 1 |  |  |  |  |  |  |  |  |  |  |  |  |
| 3 | Age^a^ | .06* | -.26*** | 1 |  |  |  |  |  |  |  |  |  |  |  |
| 4 | Gender^b^ | .11** | .12*** | -.14*** | 1 |  |  |  |  |  |  |  |  |  |  |
| 5 | City ≥ 100.000^b^ | -.01 | -.01 | .03 | -.02 | 1 |  |  |  |  |  |  |  |  |  |
| 6 | Police contacts^b^ | .22*** | -.15*** | .27*** | -.05 | .08* | 1 |  |  |  |  |  |  |  |  |
| 7 | Education level parents | -.03 | -.06 | .01 | -.06* | -.01 | .01 | 1 |  |  |  |  |  |  |  |
| 8 | GAF score^a^ | -.16*** | .30*** | -.06 | -.01 | .04 | -.10** | .06 | 1 |  |  |  |  |  |  |
| 9 | Stressful life events^b^ | .06 | -.18*** | .11** | -.05 | .04 | .10** | -.13*** | -.06 | 1 |  |  |  |  |  |
| 10 | ADHD^b^ | -.004 | .18*** | -.20*** | .11** | .04 | -.03 | -.07* | .03 | -.04 | 1 |  |  |  |  |
| 11 | ASD^b^ | .14*** | -.16*** | -.06 | .18*** | -.05 | -.05 | .06 | -.23*** | -.06 | -.27*** | 1 |  |  |  |
| 12 | Anxiety / Mood^b^ | -.13*** | -.13*** | .27*** | -.19*** | -.01 | .01 | .01 | -.09** | .10** | -.20*** | -.25*** | 1 |  |  |
| 13 | DBD-NOS / ODD^b^ | .23*** | -.13*** | .05 | .02 | .04 | .15*** | -.08* | -.13*** | .06 | .04 | -.10** | -.08* | 1 |  |
| 14 | Other Diagnoses^b^ | .04 | -.07* | .12*** | -.11*** | .06 | .10** | -.09** | -.001 | .14*** | -.18*** | -.24*** | .12*** | .03 | 1 |

* *p* < .05, ** *p* < .01, *** p < .001 (2-tailed)

^a^ continuous scores, ^b^ dichotomous scores

Table S4

*Hierarchical multiple linear regression analyses predicting Kidscreen scores from age, gender and ICU total score (continuous) by diagnosis*

|  |  | Total Group | |  | ADHD^1^ | |  | ASD^1^ | |  | Anxiety / Mood^1^ | |  | DBD-NOS / ODD^1^ | |  | Other Diagnoses^1^ | |
| --- | --- | --- | --- | --- | --- | --- | --- | --- | --- | --- | --- | --- | --- | --- | --- | --- | --- | --- |
|  |  | Δ*R^2^* | β |  | Δ*R^2^* | β |  | Δ*R^2^* | β |  | Δ*R^2^* | β |  | Δ*R^2^* | β |  | Δ*R^2^* | β |
| Step 1 |  | .076*** |  |  | .055*** |  |  | .043*** |  |  | .072*** |  |  | .104* |  |  | .123*** |  |
|  | Age |  | -.252*** |  |  | -.230*** |  |  | -.190*** |  |  | -.195** |  |  | -.323** |  |  | -.278*** |
|  | Gender |  | -.084** |  |  | -.033 |  |  | -.078 |  |  | -.153* |  |  | .012 |  |  | -.170*** |
| Step 2 |  | .097*** |  |  | .138*** |  |  | .085*** |  |  | .080*** |  |  | .127** |  |  | .104*** |  |
|  | ICU ≥ 32 |  | -.314*** |  |  | -.375*** |  |  | -.294*** |  |  | -.285*** |  |  | -.360** |  |  | -.327*** |
| Total *R^2^* |  | .173*** |  |  | .193*** |  |  | .128*** |  |  | .153*** |  |  | .231** |  |  | .227*** |  |
| *n* |  | 1.014 |  |  | 444 |  |  | 434 |  |  | 237 |  |  | 73 |  |  | 421 |  |

* *p*< .05. ** *p*< .01 *** *p*< .001

^1^ with and without comorbidity

*Note.*

β= standardized regression coefficient

ICU = Inventory of Callous-Unemotional traits

ADHD = Attention-Deficit/Hyperactivity Disorder

ASD = Autism Spectrum Disorder

Anxiety / Mood = either Anxiety or Mood disorder

DBD-NOS / ODD = either Disruptive Behavior Disorder Not Otherwise Specified or Oppositional Defiant Disorder

Other Diagnoses = diagnoses listed in table S1

Table S5

*Associations between diagnosis and risk for ICU ≥ 32 expressed as odds ratio, and prevalence of high CU scores*

| Diagnosis^1^ | ICU score | |  | OR | 95% CI | | |  | OR_adj_ | 95% CI_adj_ | | |  | ICU < 32 | | ICU ≥ 32 | |
| --- | --- | --- | --- | --- | --- | --- | --- | --- | --- | --- | --- | --- | --- | --- | --- | --- | --- |
|  | *M* | *(SD)* |  |  |  |  |  |  |  |  |  |  |  | *n* | % | *n* | % |
| ADHD | 26.4 | 9.4 |  | .62* | .42 | - | .90 |  | .60* | .41 | - | .89 |  | 120 | 72.3 | 46 | 27.7 |
| ASD | 30.1 | 10.3 |  | 1.64** | 1.18 | - | 2.27 |  | 1.57** | 1.13 | - | 2.18 |  | 113 | 55.1 | 92 | 44.9 |
| Anxiety / Mood | 22.8 | 11.3 |  | .33** | .16 | - | .69 |  | .34** | .16 | - | .72 |  | 39 | 81.3 | 9 | 18.8 |
| DBD-NOS / ODD | 30.2 | 10.7 |  | 1.29 | 1.55 | - | 4.64 |  | 1.71 | .57 | - | 5.2 |  | 7 | 53.8 | 6 | 46.2 |
| Other Diagnoses | 28.5 | 11.5 |  | .86 | .52 | - | 1.42 |  | .924 | .56 | - | 1.54 |  | 50 | 65.8 | 26 | 34.2 |

* *p* < .05; ** *p* < .01; *** *p* < .001

^1^ Without comorbidity

_adj_= Adjusted for age and gender

_adj_= Adjusted for age and gender

*Note.*

OR = odds ratio; ICU = Inventory of Callous Unemotional traits; 95%CI = 95% confidence Interval

ADHD = Attention-Deficit/Hyperactivity Disorder

ASD = Autism Spectrum Disorder

Anxiety / Mood = either Anxiety or Mood disorder

DBD-NOS / ODD = either Disruptive Behavior Disorder Not Otherwise Specified or Oppositional Defiant Disorder

Other Diagnoses = diagnoses listed in table S1

Table S6

*Mean Kidscreen scores and standard error of the mean (SEM) by diagnosis*

|  | Dx without comorbidity | | |  |  |
| --- | --- | --- | --- | --- | --- |
|  | ICU < 32 | SEM | ICU ≥ 32 | SEM | *p*-value |
| ADHD | 104.2 | 1.22 | 98.6 | 1.64 | .014 |
| ASD | 95.8 | 1.17 | 91.7 | 1.32 | .020 |
| Anxiety / Mood | 97.9 | 2.13 | 96.7 | 3.83 | .803 |
| DBD-NOS / ODD | 100.9 | 4.60 | 91.8 | 3.04 | .143 |
| Other Diagnoses | 101.9 | 1.68 | 90.2 | 2.23 | <.001 |

*Note.*

ICU = Inventory of Callous-Unemotional traits

ADHD = Attention-Deficit/Hyperactivity Disorder

ASD = Autism Spectrum Disorder

Anxiety / Mood = either Anxiety or Mood disorder

DBD-NOS / ODD = either Disruptive Behavior Disorder Not Otherwise Specified or Oppositional Defiant Disorder

Other diagnoses = diagnoses listed in table S1

Table S7

*Hierarchical multiple linear regression analyses predicting Kidscreen scores from age, gender and ICU by diagnosis*

|  |  | Total Group | |  | ADHD^1^ | |  | ASD^1^ | |  | Anxiety / Mood^1^ | |  | DBD-NOS / ODD^1^ | |  | Other Diagnoses^1^ | |
| --- | --- | --- | --- | --- | --- | --- | --- | --- | --- | --- | --- | --- | --- | --- | --- | --- | --- | --- |
|  |  | Δ*R^2^* | β |  | Δ*R^2^* | β |  | Δ*R^2^* | β |  | Δ*R^2^* | β |  | Δ*R^2^* | β |  | Δ*R^2^* | β |
| Step 1 |  | .076*** |  |  | .056** |  |  | .031* |  |  | .108 |  |  | .031 |  |  | .192*** |  |
|  | Age |  | -.252*** |  |  | -.220** |  |  | -.175* |  |  | -.269 |  |  | .123 |  |  | -.345** |
|  | Gender |  | -.084** |  |  | -.062 |  |  | -.005 |  |  | -.126 |  |  | -.097 |  |  | -.249* |
| Step 2 |  | .070*** |  |  | .057** |  |  | .026* |  |  | .003 |  |  | .184 |  |  | .135*** |  |
|  | ICU ≥ 32 |  | -.266*** |  |  | -.245** |  |  | -.162* |  |  | -.051 |  |  | -.433 |  |  | -.390*** |
| Total *R^2^* |  | .147*** |  |  | .113** |  |  | .057* |  |  | .111 |  |  | .215 |  |  | .327*** |  |
| *n* |  | 1,014 |  |  | 165 |  |  | 203 |  |  | 47 |  |  | 12 |  |  | 75 |  |

* *p*< .05, ** *p*< .01 *** *p*< .001

^1^ Without comorbidity

*Note.*

β= standardized regression coefficient

ICU = Inventory of Callous-Unemotional traits

ADHD = Attention-Deficit/Hyperactivity Disorder

ASD = Autism Spectrum Disorder

Anxiety / Mood = either Anxiety or Mood disorder

DBD-NOS / ODD = either Disruptive Behavior Disorder Not Otherwise Specified or Oppositional Defiant Disorder

Other Diagnoses = diagnoses listed in table S1
